# Social object play between captive bottlenose and Risso's dolphins

**DOI:** 10.1371/journal.pone.0196658

**Published:** 2018-05-23

**Authors:** Hisako Ikeda, Masayuki Komaba, Kumiko Komaba, Ayaka Matsuya, Akihiro Kawakubo, Fumio Nakahara

**Affiliations:** 1 Kujukushima Aquarium–Umi Kirara, Sasebo, Nagasaki, Japan; 2 Tokiwa University, Mito, Ibaraki, Japan; University of Missouri Columbia, UNITED STATES

## Abstract

Many animal species engage in social object play with movable objects. Two bottlenose dolphins (*Tursiops truncatus*) and one Risso’s dolphin (*Grampus griseus*) owned by the Kujukushima Aquarium, Japan, occasionally shared and played with an object. Herein, we report social object play between two dolphins exchanging a ball in water. Just before delivery of the ball, one dolphin made an action to request the ball from the dolphin that possessed the ball. This request behavior is also discussed in this report. This study is the first to report two different cetacean species engaging in social object play with one object.

## Introduction

Play has long been identified as a potential welfare indicator because it often disappears when animals are under fitness challenge and because it is thought to be accompanied by a pleasurable emotional experience [1]. Play behavior is often qualitatively easy to recognize but difficult to define. Burghardt [2] defined play as a behavior that is repeated; incompletely functional; and differs structurally, contextually, or ontogenetically from more serious behaviors. Play is voluntarily initiated when the animal is in a relaxed or low-stress setting. It is somewhat difficult to show that all of these criteria can be applied for animals. However, some species show creative ways of interacting with their environment that appear to serve no particular purpose, and in these cases play is an obvious explanation. Such behaviors may also provide learning opportunities or stress relief that ultimately generates a survival benefit [3].

Despite definitional deficiencies, behaviors that have been characterized as play have been observed in a diverse group of animals including reptiles, birds, invertebrates, and mammals [4]. Play behavior is generally classified into three primary categories: (1) locomotor play, i.e., a solitary activity in which an animal performs intense or sustained locomotor movements [2]; (2) object play, i.e., involvement of inanimate objects of various types in an animal’s environment [5]; and (3) social play, i.e., play that is directed at conspecifics, or other animals taking on the role (at least partially) of a conspecific [2]. Little research has been conducted on play types, particularly social object play (SOP), which features in multiple categories [6]. Young individuals of many animal species have been reported to engage in SOP with movable objects [6]. SOP is often understood in association with property ownership. Primate researchers have been interested in SOP from the perspective of object possession among monkeys: when a valuable object in limited supply is unclaimed, a dominant individual will most likely have access to it [7]. However, there is also a so-called “prior possession rule” [8] whereby individuals rarely compete directly for valuable portable objects that are already claimed by other individuals, even when object holders are subordinates [6].

Play is common among dolphins of many species, and both juvenile and mature individuals engage in play [4]. Dolphins engage in a variety of locomotor play, including aerial behaviors, erratic swimming, intentional stranding, and surfing [4]. In addition, dolphins occasionally play with objects encountered in the environment. In the wild, objects such as seaweed, wood pieces, feathers, fish, and plastic bags are used as play objects by dolphins. In captivity, dolphins often play with artificial objects such as balls, rings, tires, and pipes [9]. When available, balls appear to be a popular choice as play objects [4]. Some researchers have considered the behavior of making bubble rings as a feature of object play [10]. Social play may be more common but is also more difficult to recognize [3]. Dolphins may be engaged in social play when cooperating while playing with airborne antics, erratic swimming, chasing, and tactile interactions [4]. Although a report on multiple individuals of bottlenose dolphin (*Tursiops truncatus*) engaging in object play has been published [9], it has not been confirmed whether this behavior constitutes SOP. Dolphins may appear to be simultaneously playing with the same objects; however, to date, no action of actively relinquishing ownership has been observed. Kuczaj and Highfill [11] observed a scene in which wild rough-toothed dolphins (*Steno bredanensis*) handled plastic pieces while also chasing each other. However, based on this observation alone, it cannot be demonstrated that the dolphins were actively relinquishing ownership.

In the present study, we raised two female bottlenose dolphins and one female Risso’s dolphin (*Grampus griseus*) in the Kujukushima Aquarium, Umi-Kirara, Japan. The dolphins daily played with toys in the pool, thereby allowing us to observe various behaviors. Herein, we report SOP in which one dolphin passed the ball to others underwater and present an accompanying video. Across taxa, play behavior has not been extensively studied, and consensus about its function remains unclear [5, 12]. This study is the first to report SOP between different species with the exception of cases between human caregivers and pets. The purpose of this study was to describe this rarely reported behavior and discuss possible ecological and social factors of interspecific SOP.

## Materials and methods

### Subjects

The video was taken at the Kujukushima Aquarium, Umi Kirara, Japan on September 9, 2012. The subjects were Nami and Niha, two female bottlenose dolphins, and Lily, a female Risso’s dolphin. All dolphins were approximately 10 years old at the time of the study. The dolphins were housed in a main pool (11.5 m in diameter and 4.5 m in depth) and an adjacent holding pool (5.4 m in diameter and 3 m in depth). Since the three dolphins were brought to the aquarium in May 2009, they have had access to toys for most of the time during which the aquarium was open. The dolphins had access to approximately 50 types of toys made of different materials. We added toys in the pool at times other than during the program and training when the aquarium was open and randomized the number of toys and their combinations.

### Ethics statement

This study was conducted in compliance with the Ethical Guidelines for the Conduct of Research on Animals by Zoos and Aquariums issued by the World Association of Zoos and Aquariums (WAZA) and the Code of Ethics issued by the Japanese Association of Zoos and Aquariums (JAZA). Research permission for this study was granted by the Kujukushima Aquarium, Umi Kirara, Japan. This study was observational and did not involve handling of animals. This observation did not affect the welfare of dolphins.

### Data recording

The aquarium staff (HI, MK, KK, and AM) routinely observed dolphin behaviors in their spare time. We used a behavior sampling method in which an observer watched the entire group of subjects and recorded each occurrence of a particular type of behavior [13]. In the present study, the particular type of behavior was “ball exchange”. A digital HD video camera recorder HDR-CX590V (Sony, Japan) was used to record dolphin behaviors. Videos were mainly shot from a forward position in which the main pool was in front of the camera and the holding pool was half-visible. Video recording was conducted when the dolphins were allowed to play with two balls, and two other toys were added in the pool. When a ball moved between individuals, we recorded the owner of the ball and the individual receiving the ball.

### Definitions and recording items

For ball delivery, we considered a dolphin to be in possession of the ball when she held the ball in her mouth. The transfer of possession was recorded when the dolphin who originally possessed the ball released it from her mouth after which another dolphin held in her mouth. When possession was transferred twice in succession between two dolphins, we considered the possession as round-tripped. When possession was returned to the dolphin that moved the ball for the first time in two consecutive movements, we considered this as one round trip of possession.

Request behavior for the ball by the opponent was defined as follows: the dolphin without the ball placed her rostrum to the mouth of the ball-holder and opened her mouth before the ball left the mouth of the ball-holder. In this behavior, the two dolphins were swimming side-by-side or the rostrum of the dolphin swimming behind was between the rostrum and the pectoral fin of the dolphin swimming ahead.

## Results

Ball exchange between dolphins was observed between 16:14 and 16:21 on September 9, 2012. During the 7-min observation, 4 min and 43 s of video was recorded.

### Transfer of possession

**Fig 1:**
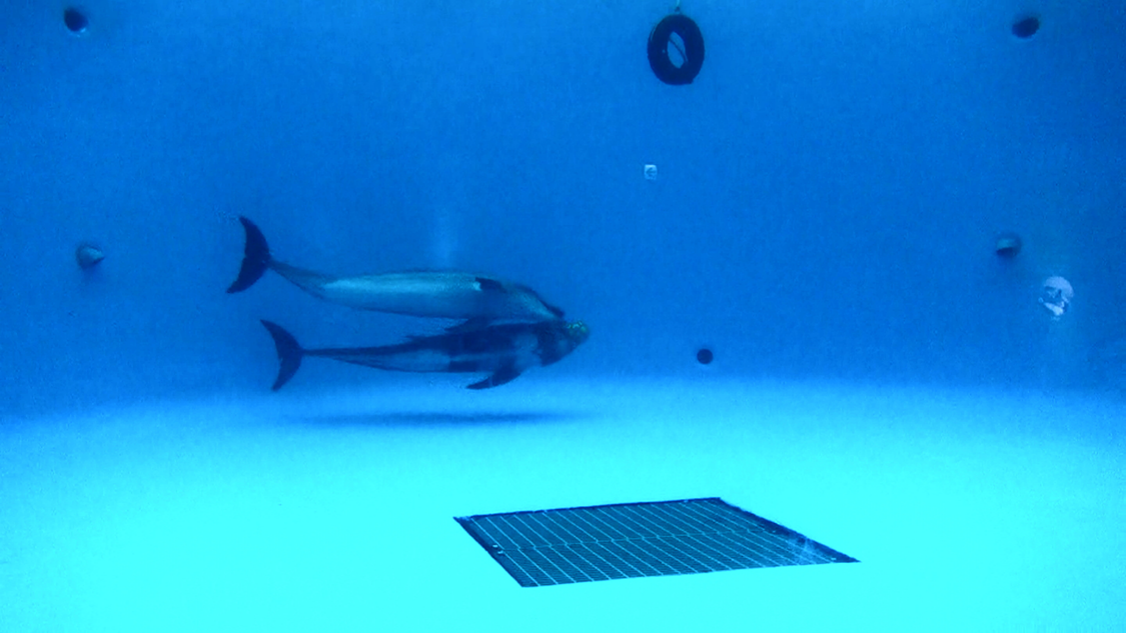
Ball exchange between a bottlenose dolphin (Niha) and a Risso’s dolphin (Lily). Lily released the ball from her mouth and Niha caught it.

**Fig 2:**
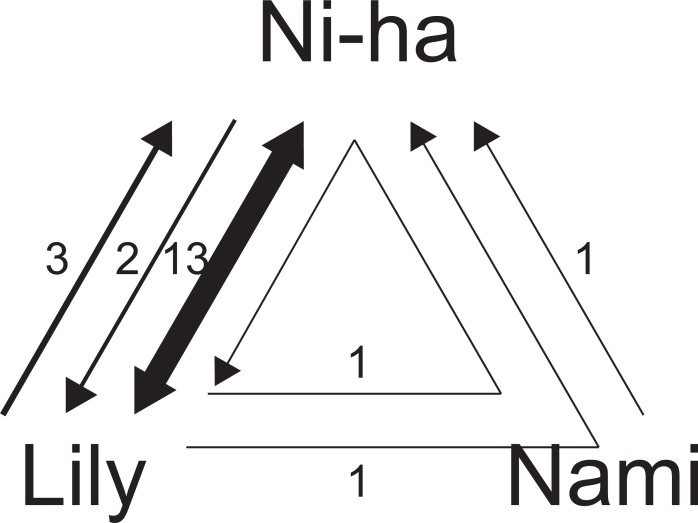
Ball movement diagram. Ball movement between the three individuals: two bottlenose dolphins (Niha and Nami) and a Risso’s dolphin (Lily). An arrow indicates the destination of the ball in each case. The number in the figure indicates the number of times the ball moved.

**Fig 3:**
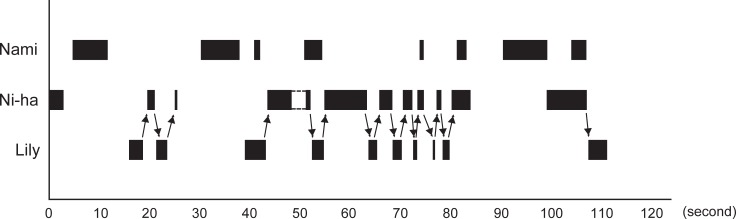
An example of a bout of social object play. The vertical axis indicates the individual, and the horizontal axis indicates time. The hatched bar is the time of possession of the ball. The arrow indicates the destination of the ball. In this figure, Nami (bottlenose dolphin) possesses the ball by herself, whereas Niha (bottlenose dolphin) and Lily (Risso’s dolphin) share the ball.

Ball movement (ownership movement) was observed 37 times during this period. Of these movements, 13 were round trips of possessions (26 transfers of possession) between Niha and Lily (Fig 1). Ball movement in one direction occurred thrice from Lily to Niha, twice from Niha to Lily, and once from Nami to Niha (Fig 2). Possession switched twice in one direction among all three individuals. In both cases, the possession moved in the same order: from Lily to Nami and finally to Niha. In one case, the ball was returned to Lily at the end (Fig 2). No transfer of possession was observed from Niha to Nami or from Nami to Lily. Fig 3 shows an example of a long bout of ball exchange behavior. The ball went back and forth seven times, and three movements occurred in one direction from Niha to Lily (S1 Video).

### Ball movement after request behavior

The ball moved 22 times out of the 26 request behaviors. We observed 12 possession changes from Niha to Lily after Lily demonstrated 16 request behaviors. We observed nine possession changes from Lily to Niha after nine request behaviors by Niha and one possession change from Nami to Niha following one request behavior by Niha. Although Nami and Lily responded to all Niha’s requests, Niha did not respond to Lily’s request behavior four times, and thus, possessions did not always change following her request behavior. No request behaviors were observed between Nami and Lily.

## Discussion

To the best of our knowledge, the present study is the first to report SOP between different species with the exception of human caregivers and pets. Of the 37 ball exchanges observed, 34 exchanges occurred between a bottlenose dolphin and a Risso’s dolphin (i.e., 32 times between Niha and Lily and 2 times between Nami and Lily). Niha and Lily spent more time swimming together than any other pair, with both identified association indices of 0.33, 0.23, and 0.08 for Niha-Lily, Niha-Nami, and Nami-Lily pairs, respectively (Nakahara, personal communication). Association levels may therefore have influenced the large number of exchanges between Niha and Lily. Thus, it appears that daily inter-individual relationships influence ball exchange behavior. Mixed-species associations of bottlenose dolphins and Risso’s dolphins have also been observed in the wild [14], and hybrid individuals are known in both captivity and the wild [15]. Given the social relationship between the two species, the occurrence of SOP is not surprising.

Dolphins are social animals that establish dominance relationships [16]. Lily was considered to be subordinate to Nami and Niha based on interactions among the three individuals. The dominance order calculated using the simple dominance index is as follows: Niha (60.6) > Nami (34.6) > Lily (4.8) (Nakahara, personal communication). In our study, repeated patterns of ball transfer occurred from subordinate dolphins to dominant dolphins and from dominant dolphins to subordinate dolphins. In primates, dominant individuals tend to keep objects of limited value if there are no requests from other individuals [6]. During ball movement from Niha to Lily, Lily’s request behaviors resulted in ball movement 75% (12/16) of the time. This suggests that in bottlenose and Risso’s dolphins, dominant individuals may relinquish possession of an object to a subordinate if a request is made.

Individuals also engage in role-reversing and self-handicapping [5] to maintain social play [17]. Each can serve to reduce asymmetries between interacting animals and foster necessary reciprocity for play to occur [17]. Reciprocity is an important component of prosocial behavior, which encompasses a broad range of activities intended to benefit one or more individuals other than oneself [18]. Yamamoto [19] summarized characteristics of prosocial behavior in chimpanzees with the following three points: (1) chimpanzees do not help spontaneously but respond to explicit requests from others, (2) they can understand the desire of others by looking at others’ situations, and (3) even if they understand others’ demands, they may not always provide voluntary help. In our study, we found that dolphins could respond to an explicit request. However, the transfer of possession occurred even if there was no clear request. This indicates the possibility of play by actively changing possession of the ball. Bottlenose dolphins perform spontaneous prosocial choices [20], and spontaneous transfer of possession may occur.

In this observation, we noticed that the individual who previously possessed the ball switched possession to another individual. The possession eventually returned to the original individual in some but not all instances. This result suggests that the mechanisms of SOP in dolphins differ from those applied in the “prior possession rule” in primates. Future research is necessary to address this point.

Object play involving multiple individuals has not been frequently reported in dolphins. In the present study, we observed SOP with active change of possession among dolphins for the first time. We could also confirm SOP for the first time between two different species of dolphins, i.e., bottlenose and Risso’s dolphins. As with chimpanzees, we confirmed that dolphins can understand the demands of others and can respond to requests. Multiple studies have been conducted on play in primates, including in humans and chimpanzees [2]. However, play among dolphins, which are also considered to be intelligent, has only recently started being investigated. By advancing research on play among dolphins, comparative studies with dolphins and chimpanzees or humans will be possible.

## Supporting information

S1 Video Social object play between captive bottlenose and Risso’s dolphins. (MP4)
